# Fast neural learning in dogs: A multimodal sensory fMRI study

**DOI:** 10.1038/s41598-018-32990-2

**Published:** 2018-10-02

**Authors:** Ashley Prichard, Raveena Chhibber, Kate Athanassiades, Mark Spivak, Gregory S. Berns

**Affiliations:** 1 Psychology Department, Emory University, Atlanta, GA 30322 USA; 2 Comprehensive Pet Therapy, Atlanta, GA 30328 USA

## Abstract

Dogs may follow their nose, but they learn associations to many types of sensory stimuli. Are some modalities learned better than others? We used awake fMRI in 19 dogs over a series of three experiments to measure reward-related learning of visual, olfactory, and verbal stimuli. Neurobiological learning curves were generated for individual dogs by measuring activation over time within three regions of interest: the caudate nucleus, amygdala, and parietotemporal cortex. The learning curves showed that dogs formed stimulus-reward associations in as little as 22 trials. Consistent with neuroimaging studies of associative learning, the caudate showed a main effect for reward-related stimuli, but not a significant interaction with modality. However, there were significant differences in the time courses, suggesting that although multiple modalities are represented in the caudate, the rates of acquisition and habituation are modality-dependent and are potentially gated by their salience in the amygdala. Visual and olfactory modalities resulted in the fastest learning, while verbal stimuli were least effective, suggesting that verbal commands may be the least efficient way to train dogs.

## Introduction

It is well known that dogs have keen sensory abilities, but are some modalities learned better than others? For example, a dog’s behavior is popularly considered to be driven by their noses^1^. On the other hand, dogs have superior hearing to humans and readily form visual associations – even being able to discriminate human facial expressions^2–4^. The experimental literature has shown that dogs can learn associations to almost any stimulus, but demonstrating that certain modalities are innately preferred over others has been difficult^5^. Apart from the basic question of how different sensory modalities impact associative learning in dogs, the answer could affect how dogs are trained in an optimal manner.

While behavioral mechanisms underlying associative learning are well-described, there has been increasing sophistication in neural methods to understand how these associations are formed in the brain. In humans, functional magnetic resonance imaging (fMRI) has become the preferred neuroscience tool because of its noninvasiveness. Coupled with computational models, this approach has been successful in parsing the contributions of different brain structures to reinforcement learning. Several fMRI studies have demonstrated that the striatum “learns” the value of visual stimuli in a manner consistent with reward-prediction error models, regardless of whether the reward is a primary taste reward or money^6–10^. Similar results have been obtained for visual cues that predict pleasant and unpleasant odors, although the time courses varied by the nature of the odor and brain region (e.g. striatum, orbitofrontal cortex, or amygdala)^11^. More generally, the amygdala has been hypothesized to interact with the reward-learning process by gating attention to salient stimuli^12,13^.

Like humans, dogs can be trained for non-invasive fMRI studies^14^. Early dog-fMRI studies demonstrated the replicability and reliability of caudate activation in response to hand signals predictive of food reward^15^. Later studies extended these results and showed that caudate and amygdala activation were correlated with specific aspects of a dog’s temperament and could even be used as part of a biometric predictor for suitability as a service-dog^16^. Although initial studies relied on visual signals, later work suggested that both olfactory and verbal cues (e.g. social praise) could also elicit activity in the caudate^17,18^.

Here, we used fMRI to measure the neural rates of associative learning in dogs to three modalities: visual, olfactory, and verbal. In three separate scanning sessions, each devoted to one modality, dogs were presented with two stimuli they had never encountered before. During each scan session, one of the stimuli (the conditioned stimulus) was always followed by a food reward, and the other (the control stimulus) nothing. If dogs formed modality-independent associations between the conditioned stimuli and reward, activity in the caudate nucleus should increase over time in response to the conditioned reward stimulus relative to the control stimulus, regardless of the modality. Similarly, if the amygdala functions as an attentional gate to learning, stimuli that are most salient to a dog (e.g. odorants) would result in greater activation in this structure. Lastly, if dogs preferentially process learning associations in one stimulus modality over another, then there will be a difference in the neural rate of learning between the three modalities.

## Materials and Methods

### Participants

Participants were 19 pet dogs volunteered by their Atlanta owners for fMRI training and fMRI studies^14,15,18,19^. All dogs had previously completed one or more scans for the project and had demonstrated the ability to participate in awake fMRI scans. The study utilized previously neutral stimuli and no physical or chemical restraint was implemented. This study was performed in accordance with the recommendations in the Guide for the Care and Use of Laboratory Animals of the National Institutes of Health. The study was approved by the Emory University IACUC (Protocols DAR-2002879-091817BA and DAR-4000079-ENTPR-A), and all owners gave written consent for their dog’s participation in the study.

### Experimental Design

Dogs entered and stationed themselves in custom chin rests in the scanner bore. All scans took place in the presence of the dog’s primary owner, who stood throughout the scan at the opening of the magnet bore, directly in front of the dogs, and delivered all rewards (hot dogs) to the dog. The owner was present to minimize any anxiety that the dog may experience due to separation, consistent with studies involving pets or human infants. An experimenter was stationed next to the owner, out of view of the dog. The experimenter controlled the timing and presentation of stimuli to the owners and the dogs via a four-button MRI-compatible button box. Onset of each stimulus was timestamped by the simultaneous press of the button box by the experimenter. Manual control of the stimuli by the experimenter was necessary, as opposed to a scripted presentation, because of the variable time it takes dogs to consume food rewards.

In three separate scanning sessions on different days, each devoted to one modality, dogs were presented with two stimuli they had never encountered before. In each session dogs were presented with either two objects, two odors, or two spoken words. All dogs completed the scan sessions in the same order (objects, odors, words), and all data collection for one modality was completed for all dogs prior to any data collection with the next modality. An event-based design was used, consisting of reward or no-reward trial types, where one stimulus within a modality was associated with the receipt of reward and the other stimulus with no-reward. On reward trials, the selected stimulus was presented for a fixed duration, which was followed by the delivery of a food reward. During no-reward trials, the second stimulus was presented for the same fixed duration and was followed by nothing. Trials were separated by an inter-trial interval specific to each modality as described below, and each dog received the same trial sequence.

Each scan session consisted of 4 runs, lasting approximately 9 minutes per run. Each run consisted of 22 trials (11 reward, 11 no-reward) with a semi-randomized presentation order, for a total of 88 trials per scan session. No trial type was repeated more than 4 times sequentially, as dogs could habituate to the stimulus, or may have a higher probability of exiting the scanner if a reward had not been issued recently. Following each run, dogs would exit the scanner and relax, drink water, or stay in the scanner to complete the next run.

Scanning was conducted with a Siemens 3 T Trio whole-body scanner using procedures described previously^14,15^. During the first of the three scans sessions, a T2-weighted structural image of the whole brain was acquired using a turbo spin-echo sequence (25–36 2 mm slices, TR = 3940 ms, TE = 8.9 ms, flip angle = 131°, 26 echo trains, 128 × 128 matrix, FOV = 192 mm). The functional scans used a single-shot echo-planar imaging (EPI) sequence to acquire volumes of 22 sequential 2.5 mm slices with a 20% gap (TE = 25 ms, TR = 1200 ms, flip angle = 70°, 64 × 64 matrix, 3 mm in-plane voxel size, FOV = 192 mm). Slices were oriented dorsally to the dog’s brain (coronal to the magnet, as in the sphinx position the dogs’ heads were positioned 90 degrees from the prone human orientation) with the phase-encoding direction right-to-left. Sequential slices were used to minimize between-plane offsets from participant movement, while the 20% slice gap minimized the “crosstalk” that can occur with sequential scan sequences. Four runs of up to 400 functional volumes were acquired for each subject, with each run lasting about 9 minutes.

### Visual Stimuli

**Figure 1:**
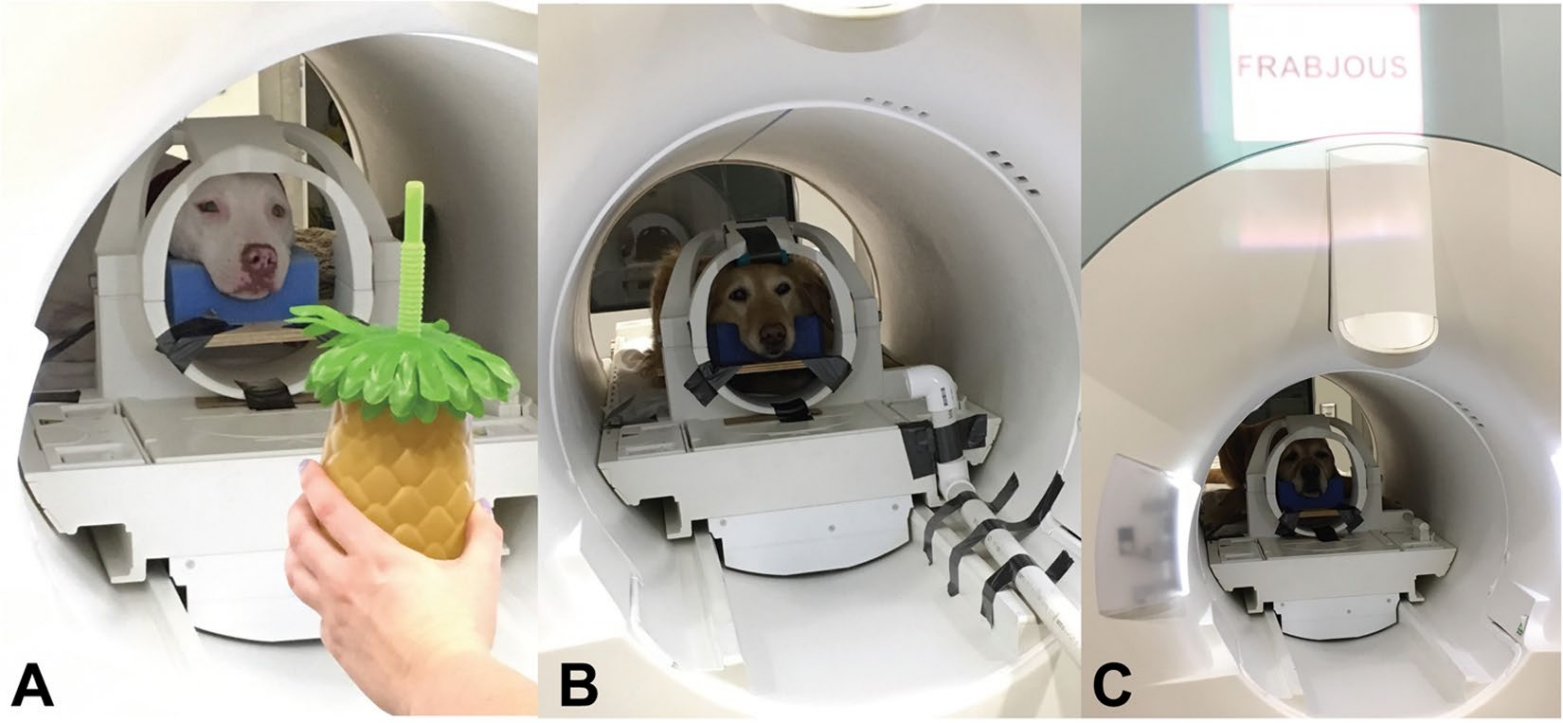
Experimental design with conditioned stimuli. Two novel stimuli were repeatedly presented during three scanning sessions, each devoted to one stimulus modality. One stimulus was associated with food (Reward), one associated with nothing (No Reward). (**A**) Presentation of pineapple object by owner to dog in MRI bore during visual modality session. (**B**) Presentation of odorants to dog in MRI bore via experimenter-controlled olfactometer during olfactory modality session. The owner remained in front of the dog. (**C**) Presentation of pseudoword *Frabjous* to owner projected above MRI bore opening during verbal modality session. The owner spoke the projected word five times per trial.

A plastic pineapple and an inflatable flamingo were used (Fig. 1A). Based on owner responses, no dog had experience with the objects prior to the scan. One object was presented at a time, held at the dog’s eye level directly at the opening of the bore for 8 s, followed by delivery of a reward (hot dog) or nothing. Trials were separated by a 7 s inter trial interval. Dogs were semi-randomly assigned the pineapple or the flamingo as the reward stimulus such that roughly half of the dogs were assigned to each group (see Table 1).

**Table 1:** Dogs (N = 19) and stimuli paired with reward.

Dog	Breed	Sex	Reward Object	Reward Odor	Reward Word
BhuBo	Boxer mix	M	Pineapple	hexanol	Callooh
Caylin	Border collie	F	Pineapple	hexanol	Frabjous
Daisy	Pitbull mix	F	Flamingo	hexanol	Callooh
Eddie	Labrador Golden mix	M	Pineapple	isoamyl acetate	Callooh
Kady	Labrador	F	Flamingo	hexanol	Frabjous
Koda	Pitbull mix	F	Flamingo	isoamyl acetate	Callooh
Libby	Pitbull mix	F	Pineapple	hexanol	Callooh
Mauja	Cattle dog mix	F	Pineapple	hexanol	N/A
Ninja	Cattle dog mix	F	Flamingo	isoamyl acetate	Frabjous
Ohana	Golden Retriever	F	Pineapple	hexanol	Frabjous
Ollie	Border collie Beagle mix	M	Flamingo	isoamyl acetate	Frabjous
Ozzie	Bichon-Yorkie mix	M	Flamingo	isoamyl acetate	Frabjous
Pearl	Golden Retriever	F	Pineapple	hexanol	Frabjous
Tallulah	Cattle Dog mix	F	Flamingo	hexanol	Callooh
Truffles	Pointer mix	F	Pineapple	isoamyl acetate	Frabjous
Tug	Portuguese Water dog	M	Flamingo	hexanol	Callooh
Velcro	Viszla	M	Pineapple	isoamyl acetate	Frabjous
Wil	Australian Shepherd	M	Pineapple	isoamyl acetate	Callooh
Zen	Labrador Golden mix	M	Flamingo	isoamyl acetate	Callooh

Dog’s names, breed, sex, and stimuli (S+) are listed.

### Olfactory Stimuli

Olfactory stimuli were aqueous solutions of isoamyl acetate (IA) and hexanol (Hex) calculated to result in approximately 5 ppm in the headspace of the container. Partial vapor pressures were calculated based on the molecular weight and reported vapor pressures of 4 mmHg and 0.9 mmHg respectively, obtained from PubChem (pubchem.ncbi.nlm.nih.gov). The odorants were miscible with water and the partial pressure of the odorant was the product of the pure odorant vapor pressure and the mole fraction of the odorant. The final dilutions in water were 0.15 mL/L for IA and 0.55 mL/L for Hex.

Odorants were delivered via a stream of air from an aquarium grade air pump (EcoPlus Commercial Air Pump 1030 GPH) through a Drierite filter (drierite.com), and afterwards through a 3-way plastic splitter to two plastic 100 mL jars containing 50 ml of odorant solutions and one jar containing 50 ml of water to serve as a control. Each solution mixed with a continuous air stream. Plastic valves were used to control directional flow of odorized air through 10′ of 1/8″ ID Teflon tube, where the mixture (air dilution of the odorant) exited a PVC tube with a 1″ diameter opening positioned in the MRI bore 12″ from the dog’s snout (Fig. 1B). The third tube carrying air from the control jar remained open throughout the presentations of odorized air, maintaining a steady air stream presented to the dog and assisting in the clearing of lingering odor within the magnet bore. Dogs were presented an odor for an initial 3.6 s during a span of 7.2 s, followed by a reward (hot dog) or nothing, with a 9.6 s inter trial interval between odor presentations. The inter trial interval was increased compared to the visual stimulus scans to ensure that the odorant within the magnet bore had cleared prior to the next trial. Dogs were semi-randomly assigned IA or Hex as the reward stimulus such that roughly half of the dogs were assigned to each group (see Table 1).

### Verbal Stimuli

Verbal stimuli were the words “Callooh” and “Frabjous” from the Lewis Carroll poem, “Jabberwocky.” The words were chosen as novel pseudowords to the dog. The words were spoken by the dog’s primary owner, who was positioned in front of the dog at the opening of the magnet bore. Both owners and dogs wore ear plugs, reducing scanner noise by 30 decibels, but allowing for intelligible speech over the scanner noise. The words were intelligible to the experimenters, who also wore ear plugs while next to the MRI during scanning, as well as the human operators in the control room through the intercom. At the start of each trial, a word was presented to the owners via a mirror relay system that projected the words onto the surface of the scanner, directly over the owner’s head (Fig. 1C). Owners were positioned in front of the dog and repeated the words five times for an average duration of 6 s. Words were repeated to ensure the dogs heard them. Words associated with reward were followed by a 4 s delay, then the delivery of a food reward, and words not associated with a reward were followed by nothing. The words were followed by a delay after their presentation for three reasons. First, a previous imaging study by our lab where dogs were presented with spoken words by their owners in the MRI showed that dogs may move initially upon hearing words, likely due to their familiarity with verbal commands, resulting in loss of data for those instances. This was also the reasoning behind the repetition of the verbal stimuli. Second, the verbal stimuli could be delivered at a much faster rate than the presentation of the objects. The rate of the delivery of the odor stimuli through the olfactometer was also dependent on the manual operation of the olfactometer by the experimenter and the length of the tube carrying scented air from the olfactometer jar to the dog’s nose. Third, if there was no delay, the timing of the BOLD response following the verbal stimuli would peak at the moment that the reward or nothing was delivered following the last repetition, resulting in additional loss of data due to movement. Trials were separated by an 8.4 s inter trial interval. Dogs were semi-randomly assigned “Frabjous” or “Callooh” as the reward stimulus such that roughly half of the dogs were assigned to each group (see Table 1). Dog Mauja was deaf, and so did not participate in the verbal stimuli experiment. Dog Libby had excessive motion in this experiment and was not included in the analysis for this stimulus modality.

### Statistical Analyses

#### Preprocessing

Preprocessing of the fMRI data included motion correction, censoring, and normalization using AFNI (NIH) and its associated functions. Two-pass, six-parameter rigid-body motion correction was used based on a hand-selected reference volume for each dog that corresponded to their average position within the magnet bore across runs. Aggressive censoring removed unusable volumes from the fMRI time sequence because dogs can move between trials, when interacting with the object, smelling an odor, hearing a word, and when consuming rewards. Data were censored when estimated motion was greater than 1 mm displacement scan-to-scan and based on outlier voxel signal intensities. Smoothing, normalization, and motion correction parameters were identical to those described in previous studies^18^. The Advanced Normalization Tools (ANTs) software was used to spatially normalize the mean of the motion-corrected functional images^20^ to the individual dog’s structural image.

#### General Linear Model

Each subject’s motion-corrected, censored, smoothed images were analyzed within a general linear model (GLM) for each voxel in the brain using 3dDeconvolve (part of the AFNI suite). Motion time courses were generated through motion correction, and constant, linear, quadratic, cubic, and quartic drift terms were included as nuisance regressors. Drift terms were included for each run to account for baseline shifts between runs as well as slow drifts unrelated to the experiment. Task related regressors for each experiment were modeled using AFNI’s dmUBLOCK and stim_times_IM functions and were as follows: (1) reward stimulus; (2) no-reward stimulus. The function creates a column in the design matrix for each of the 88 trials, allowing for the estimation of beta values for each trial. The reason for this approach was that even though the motion censoring flagged problematic volumes, it is possible that spin-history effects could result in spurious levels of activation in specific regions of interest that, when averaged over an entire run, could still affect beta estimates. Trials with beta values greater than an absolute three percent signal change were removed prior to analyses (assuming that these were beyond the physiologic range of the BOLD signal). As described next, we used the trial-by-trial betas to estimate trimmed-means from the remaining beta values.

#### Region of Interest (ROI) Analysis

**Figure 2:**
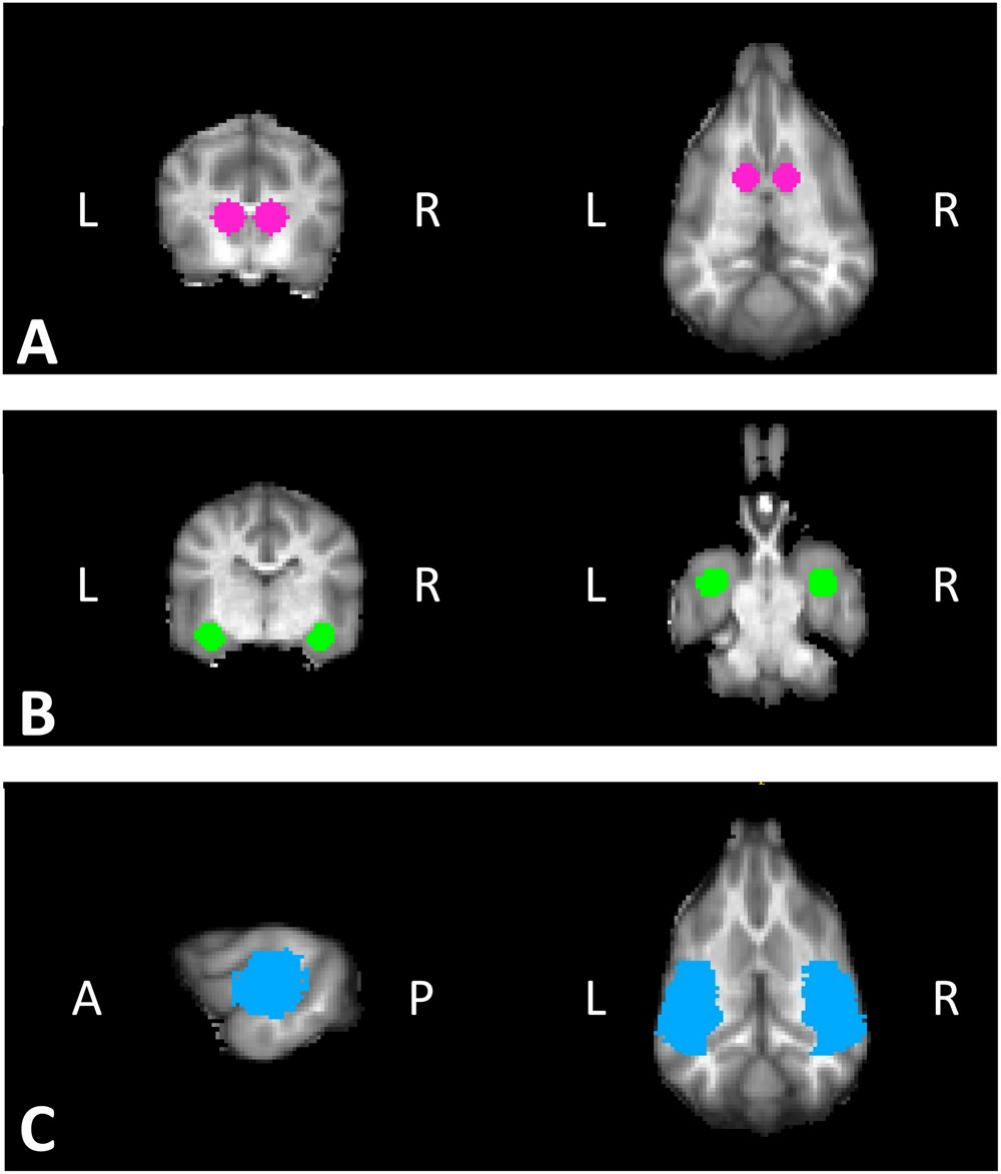
Regions of interest (ROIs) defined a priori. ROIs were drawn in individual anatomical space, example ROIs shown in template space here in transverse and dorsal views. (**A**) Caudate nuclei have been shown to differentially respond to stimuli associated with reward and no-reward. (**B**) Amygdalae have shown differential responding to stimuli associated with reward and no-reward, as well as arousal. (**C**) Parietotemporal regions including primary auditory cortex respond to verbal stimuli, including nonwords. ROI is shown here in sagittal and dorsal views in template space.

As our interest was based on the dog’s changing response to novel visual, olfactory, or verbal stimuli, all quantitative analyses based on the imaging results used activation values in the canine brain area previously observed to be responsive to visual^18^, olfactory^17,21^, and verbal^22^ stimuli. Anatomical ROIs of the left and right caudate nuclei, and the left and right amygdala were defined structurally using each dog’s T2-weighted structural image of the whole brain. A parietotemporal region was also included because of its known involvement with verbal and visual stimuli in dog fMRI studies but no reported involvement with stimulus valuation^22^. The parietotemporal region of interest was defined using a high-resolution canine brain atlas^23^ and applyANTSTransformation (part of the ANTS suite) to transform the left and right parietotemporal ROIs from template to individual space (Fig. 2). Thereafter, all analyses were performed in individual, rather than group space.

Beta values for each presentation of reward stimuli (44 trials) and no-reward stimuli (44 trials) were extracted from and averaged over the ROIs in the left and right hemispheres. Beta values were used to construct a learning curve across presentations of the stimuli by ROI, run, and modality, as well as to test for any hemispheric differences. We used the mixed-model procedure in SPSS 24 (IBM) with fixed-effects for the intercept, run number, type (reward or no-reward), modality (visual, olfactory, & verbal), ROI (amygdala, caudate, & parietotemporal), and hemisphere (left or right), identity covariance structure, and maximum-likelihood estimation. Run was modeled as a fixed effect because it made no assumptions about the time course. As hemisphere did not account for a significant amount of variance, all analyses removed hemisphere as a factor.

## Results

We found neural evidence for differentiation of the reward and no-reward stimuli in all modalities (*p* < 0.001) (Table 2). Although the amplitude of this difference varied by ROI (*p* = 0.014), there was only a marginally significant interaction with modality (*p* = 0.045). However, the modality significantly affected the temporal pattern of the difference between reward and no-reward stimuli across Run (*p* = 0.006).

**Table 2:** Model results for Reward vs. No Reward, Run, Modality, and ROI.

Fixed Effects	Numerator df	Denominator df	*F*	*Sig*.
Intercept	1	17.757	11.105	0.004
Run	3	23706.431	4.801	0.002
Rew_NoRew	1	23696.466	35.034	0.000
Modality	2	23192.742	10.798	0.000
ROI	2	23704.765	33.667	0.000
Run * Rew_NoRew	3	23692.763	3.359	0.018
Run * Modality	6	23703.114	2.794	0.010
Run * ROI	6	23690.984	2.072	0.053
Rew_NoRew * Modality	2	23695.363	3.102	0.045
Rew_NoRew * ROI	2	23690.649	4.284	0.014
Modality * ROI	4	23701.672	5.389	0.000
Run * Rew_NoRew * Modality	6	23693.671	3.039	0.006
Run * Rew_NoRew * ROI	6	23690.457	0.423	0.864
Run * Modality * ROI	12	23691.077	0.827	0.623
Rew_NoRew * Modality * ROI	4	23690.700	0.537	0.709
Run * Rew_NoRew * Modality * ROI	12	23690.415	0.461	0.938

**Figure 3:**
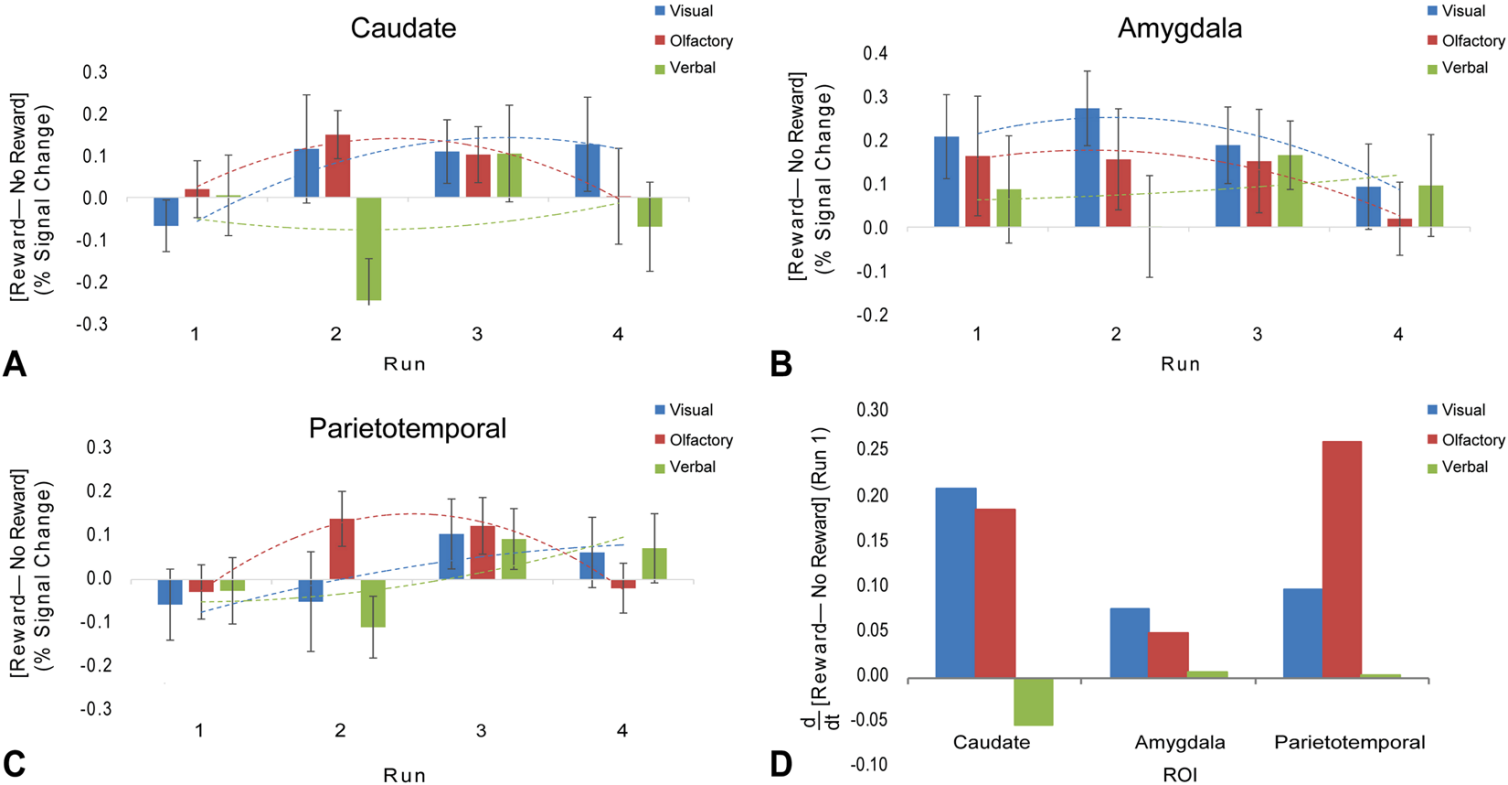
Percent signal change by ROI for the contrast of stimuli predicting Reward vs. No Reward. Unadjusted mean values across dogs by run and by modality (*blue* = visual, *red* = olfactory, *green* = verbal). Error bars denote the standard error of the mean across dogs for each modality and run. Lines denote second-order polynomial trend lines across all runs for each modality and ROI. Consistent with studies of reward learning, there were main effects of [Reward-No Reward] across all ROIs (*p* < 0.001), which was only marginally significantly different by modality (*p* = 0.045). There was a significant interaction between [Reward—No Reward] and ROI (*p* = 0.014), suggesting the magnitude of the effect was different in each region. All ROIs showed evidence of varying time course (*p* = 0.018), which differed by modality (*p* = 0.006), consistent with different rates of learning and habituation by modality. (**A**) Averaged beta values in the caudate show marked learning curves for visual and olfactory stimuli. (**B**) Averaged beta values in the amygdala show learning curves across all stimulus modalities, but verbal stimuli peak later than visual and verbal stimuli. (**C**) Averaged beta values in the parietotemporal area show weak learning effects for all modalities. (**D**) Comparison of initial learning rates for each modality for Run 1. Bars denote the temporal derivative (d/dt) of the polynomial fit for [Reward—No Reward] by modality and ROI. Across all three ROIs, percent signal change to visual and olfactory stimuli occur at a faster rate than verbal stimuli, and is evident in the first few exposures.

As there was differentiation of the reward and no-reward stimuli in all modalities, we used post-hoc analyses to examine whether these differences remained when segregated by ROI and a Bonferroni correction for multiple comparisons. In the caudate (Fig. 3A), there was a significant main effect of [Reward – No Reward] (*p* = 0.013) but not the interaction with modality (*p* = 0.081), consistent with general reward processing. There was no interaction with [Reward – No Reward] and Run, but the interaction of [Reward – No Reward] x Run x Modality was significant (*p* = 0.018), indicating that the time course of the differentiation of value varied by modality. For the caudate, both the visual and olfactory stimuli showed a rising differentiation by Runs 2 & 3, with some decrement by run 4 for olfaction.

A similar, more pronounced, pattern was observed in the amygdala (Fig. 3B). Like the caudate, the amygdala displayed a significant main effect of [Reward – No Reward] (*p* < 0.001) but no interaction with modality (*p* = 0.238). There was not a significant interaction of Run x [Reward – No Reward] (*p* = 0.584), indicating that the amygdala “learned” the differential values of the stimuli in Run 1 and maintained them throughout each experiment. Unlike the caudate, there was not a significant interaction of [Reward – No Reward] x Run x Modality (*p* = 0.707), indicating that the modality did not affect the rate of learning or habituation.

Finally, the parietotemporal cortex (Fig. 3C) also showed a main effect for [Reward – No Reward] (*p* = 0.021), but this was of marginal significance and would not survive Bonferroni correction for three separate analyses.

In sum, the neural learning curves showed that dogs formed stimulus-reward associations in as little as 22 trials. However, there were significant differences in the time courses, suggesting that the rates of acquisition and habituation were modality-dependent, with visual and olfactory modalities resulting in the fastest learning (Fig. 3D), while verbal stimuli were least effective.

## Discussion

In three experiments, we demonstrated the use of fMRI in dogs to compare associative reward-learning in the brain across visual, olfactory, and verbal modalities. Consistent with reward learning in neuroimaging studies, the caudate showed main effects for reward-related stimuli but not a significant interaction with modality. However, there were significant differences in the time courses, suggesting that although multiple modalities are represented in these structures, the rates of acquisition and habituation are modality-dependent. Further, we demonstrate that dogs have neural mechanisms that support a bias for learning conditioned visual and olfactory stimuli more rapidly than verbal stimuli.

While many fMRI studies have shown that the striatum differentially responds to conditioned stimuli associated with reward, this is the first fMRI study that directly compares reward learning across three modalities in the same participants. The significant differential effect for reward versus no-reward across multiple ROIs suggests that reward regions of the canine brain such as the striatum process the value of conditioned stimuli regardless of modality. Post-hoc analyses revealed that the primary structures associated with the differentiation of value between conditioned stimuli were the caudate and amygdala, not the parietotemporal region. Moreover, the differentiation of value was more pronounced for visual and olfactory stimuli. Interestingly, the parietotemporal cortex, which was originally selected because of its known involvement with visual and auditory stimuli, turned out to have the strongest effect for visual and olfactory stimuli. This multimodal activation can be attributed to inclusion of both primary auditory and parietal cortices within the defined region.

Although it is debatable whether the amygdala should be considered part of the “reward” circuit, its role in associative learning is well-established. One recent model suggests that the amygdala computes the surprisingness of stimuli while the striatum computes reward prediction errors^12^. A hallmark of this model is that surprise declines with repeated exposure while prediction errors remain constant as long as the stimuli themselves are unpredictable. This is exactly the pattern we observed in the caudate and the amygdala (Fig. 3), which appeared largely independent of modality. Within this framework, the amygdala activation can be interpreted as an attentional “gate” that signals the salience of a stimulus, setting up the reward system to compute its value. Further insight is gained by examining the time courses of activation in these regions.

Our results show that dogs acquired the reward associations with odors and visual stimuli at a different time course than verbal stimuli. The neural activation for visual and olfactory stimuli within the caudate and amygdala peaked by the second run, indicating the conditioned associations were formed within 22 trials. This is inconsistent with dog behavioral studies, which require days to form the stimulus-reward associations of visual or odor stimuli to reach a behavioral criterion^24–26^. However, our findings are consistent with human fMRI studies, where word learning was reported to occur at a slower rate during associative learning than visual learning^27^.

The effects of stimulus modality on differential neural time courses highlight the potential implications for training dogs. Most training protocols for dogs use gestural and verbal commands. While optimal for humans, these protocols may not be the most effective for learning from a dog’s perspective. Our results are consistent with previous behavioral findings that suggest dogs prioritize gestures over verbal commands when presented with conflicting signals^5,28^. Effective processing of visual information is essential to the social success and safety of the dog. Dogs frequently use body language as a principal mode of dog-dog communication. Tail wagging, facial expressions, and body postures are obvious examples^29–34^. In addition to visual cues, dogs use odors as a means for gaining social information from both humans and dogs^35,36^. When olfactory information is present and relevant, the dog may consider olfactory sensory information prepotent over visual information^37^. Although dogs may attend to verbal stimuli, olfactory and visual stimuli likely have greater importance in the dog’s assessment of its physical and social environment and when interacting within such environments. Our results, showing greater salience for olfactory and visual stimuli in the amygdala, are concordant with the dogs’ behavioral preferences in their natural surroundings.

There are several limitations to our study. First, although we isolated the salient modality in three separate experiments, the presence of the human owner was constant. Because the human was not blind to the nature of the stimuli, they could have inadvertently influenced the associative process through body language. However, because the olfactory modality was the most effective in eliciting reward-associations across all ROIs, and the olfactory stimuli were least likely to be picked up by the humans and were not saliently communicated by human owners, as were the display of the visual objects or the vocalization of the auditory stimulus, so-called ‘Clever Hans’ effects are unlikely to explain these results. Second, although the verbal stimuli were the least effective in forming reward-associations, this may have more to do with the discriminability of words in the scanner environment. Although the words were distinguishable to the experimenters over the scanner noise, it may have been more difficult for the dogs. There is some evidence that dogs can discriminate between spoken words during an fMRI scan, as a previous study where owners spoke trained words and pseudowords to their dogs during scanning showed neurobiological evidence that dogs were differentiating between the words in primary auditory cortices and the parietotemporal cortex^38^. This and previous results suggest some mechanistic similarity between humans and dogs for the rate of associative learning of verbal stimuli relative to other modalities. Third, we found only a marginally significant interaction between reward and modality (*p* = 0.045). Given the large sample size and high number of observations, we conclude that this is probably not a significant effect, especially since the other effects had markedly smaller *p*-values. Even so, a non-significant result does not mean that the effect doesn’t exist. It is possible that the modality of the conditioned stimuli affected the magnitude of the representation in reward-related structures like the caudate and amygdala. Undoubtedly the differential value of stimuli would be influenced by their discriminability, and as already noted, verbal cues were at a disadvantage. Fourth, the effects of habituation counteract those of learning. Habituation was perhaps most evident in the amygdala, which displayed a generally declining response with run, regardless of the modality. There is ample evidence that the amygdala habituates to repeated presentations of the same stimuli^39–41^. It would not be surprising that repeated presentation of the stimuli could lead to decreased physiological response, especially to odors. Most dogs included in the study also had experience from previous fMRI studies with conditioned object-reward associations, and some with conditioned word-object associations, such that odors within the scanner environment may have been more novel than other stimulus modalities. Finally, the stimulus-reward associations were acquired through a passive task in the scanner. No behavioral tests were conducted to test acquisition of the learned associations or to compare to the neural activations.

In summary, our results show that associative learning may be measured across multiple modalities in the caudate and that stimulus salience is denoted by the amygdala. However, certain modalities – notably visual and olfactory – were more effective in eliciting reward-related responses, especially in the rate at which they were acquired. Our results suggest that the human inclination for verbal communication appears to be based on human preferences, rather than the dog’s innate aptitude. Consequently, pet and working dog training programs would likely become more productive, with accelerated learning rates for the dog, if commands were introduced via hand signals or other physical modes of communication.
